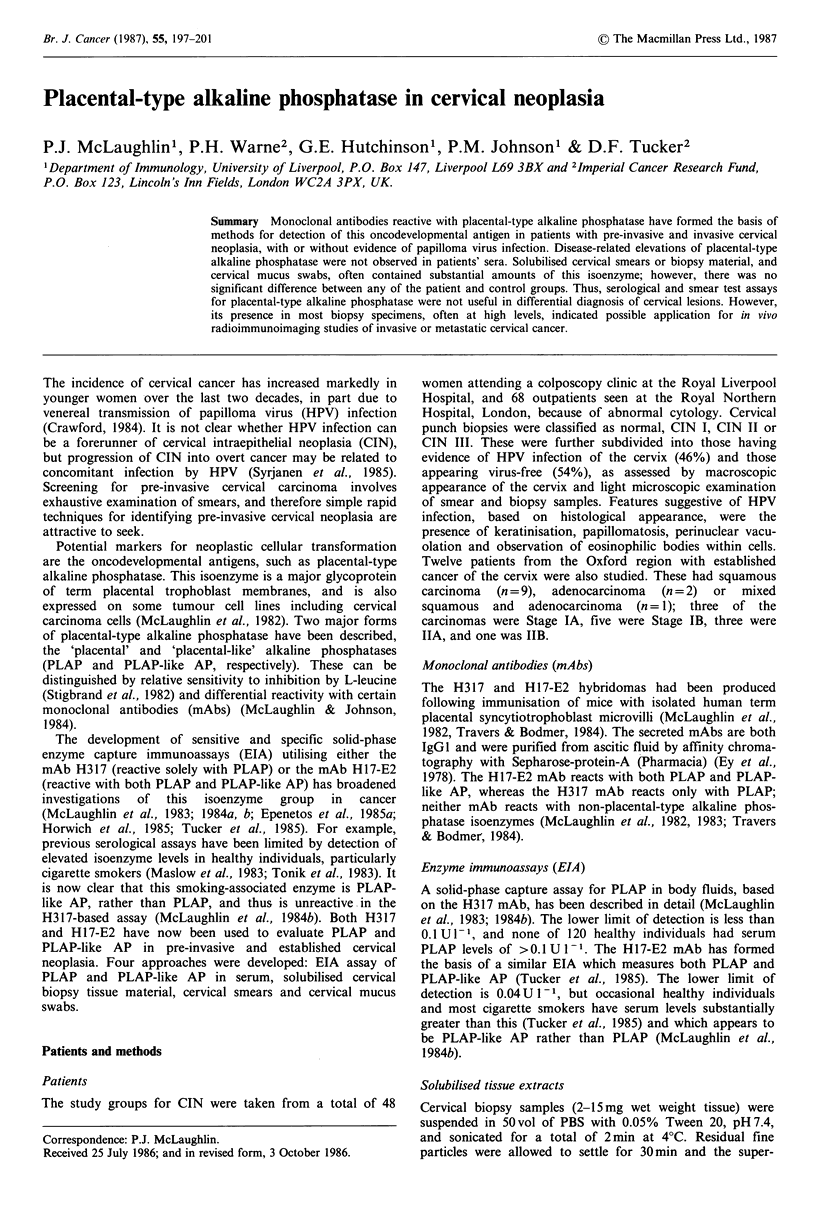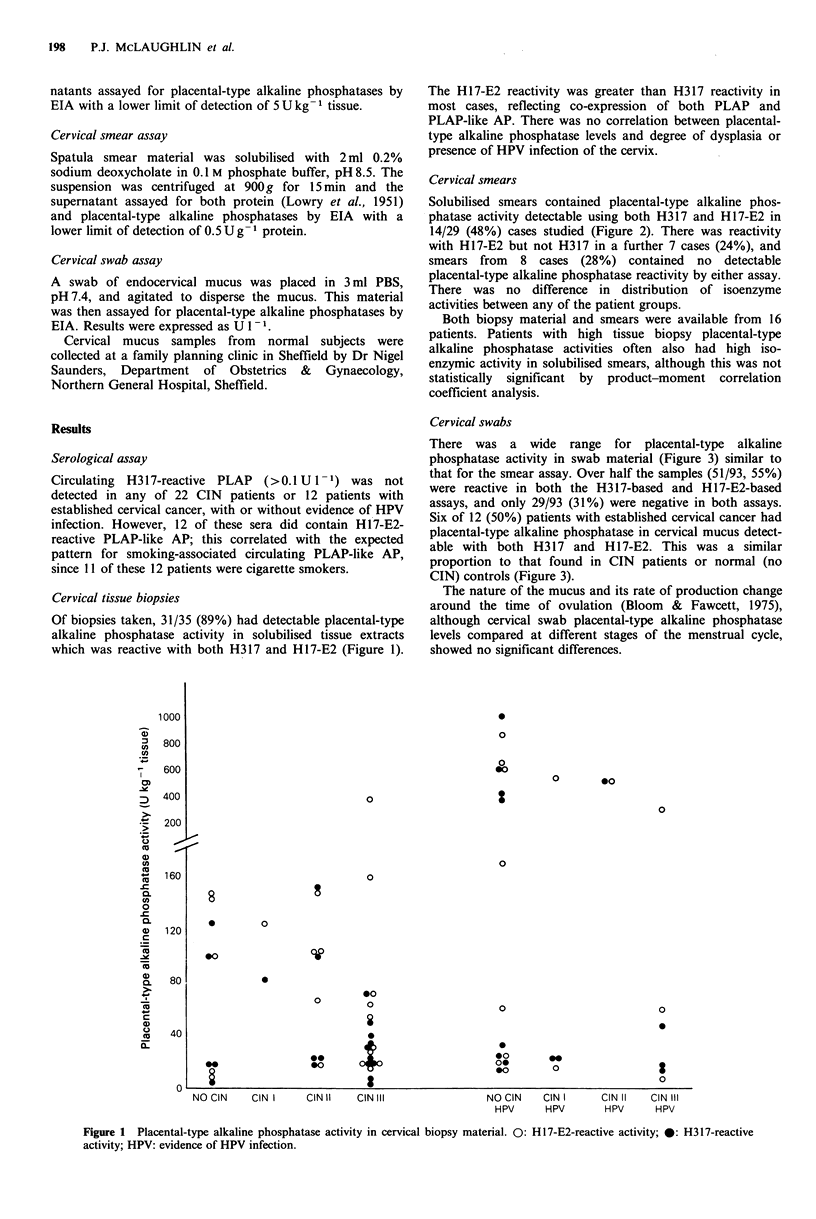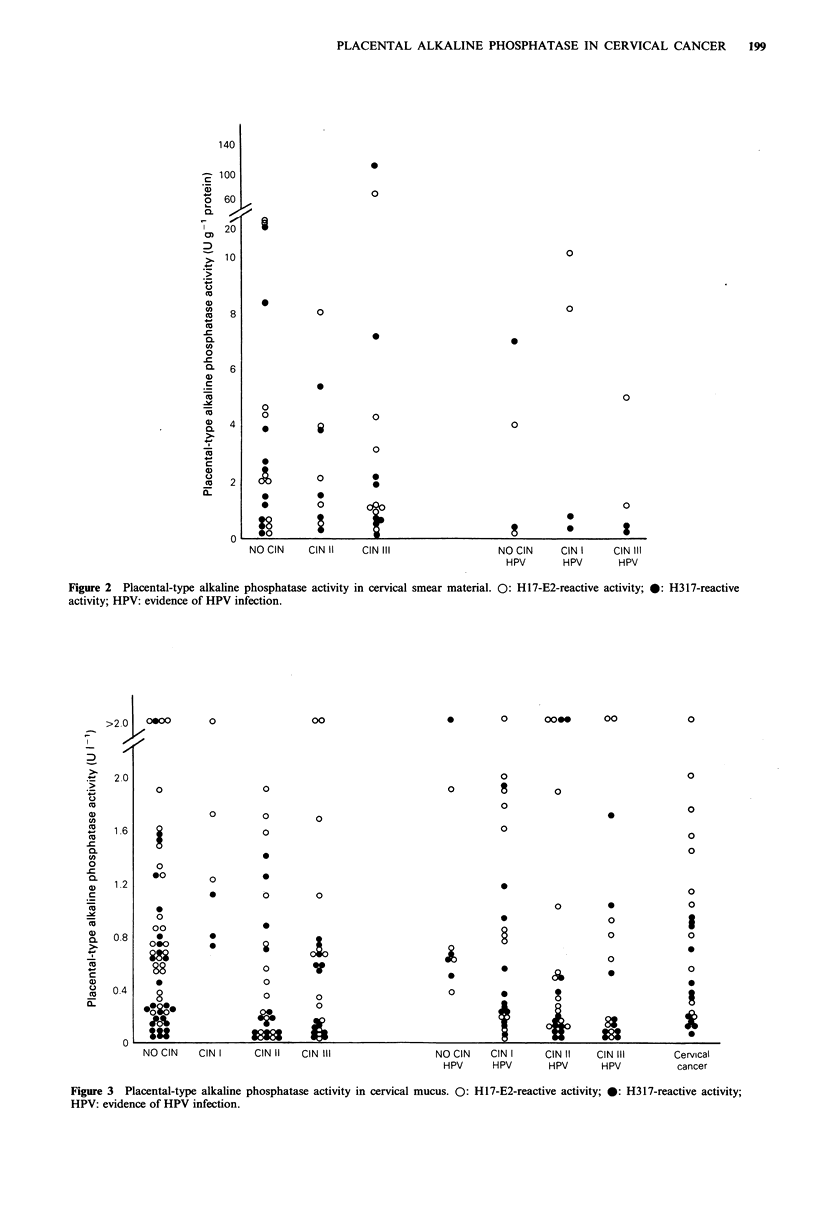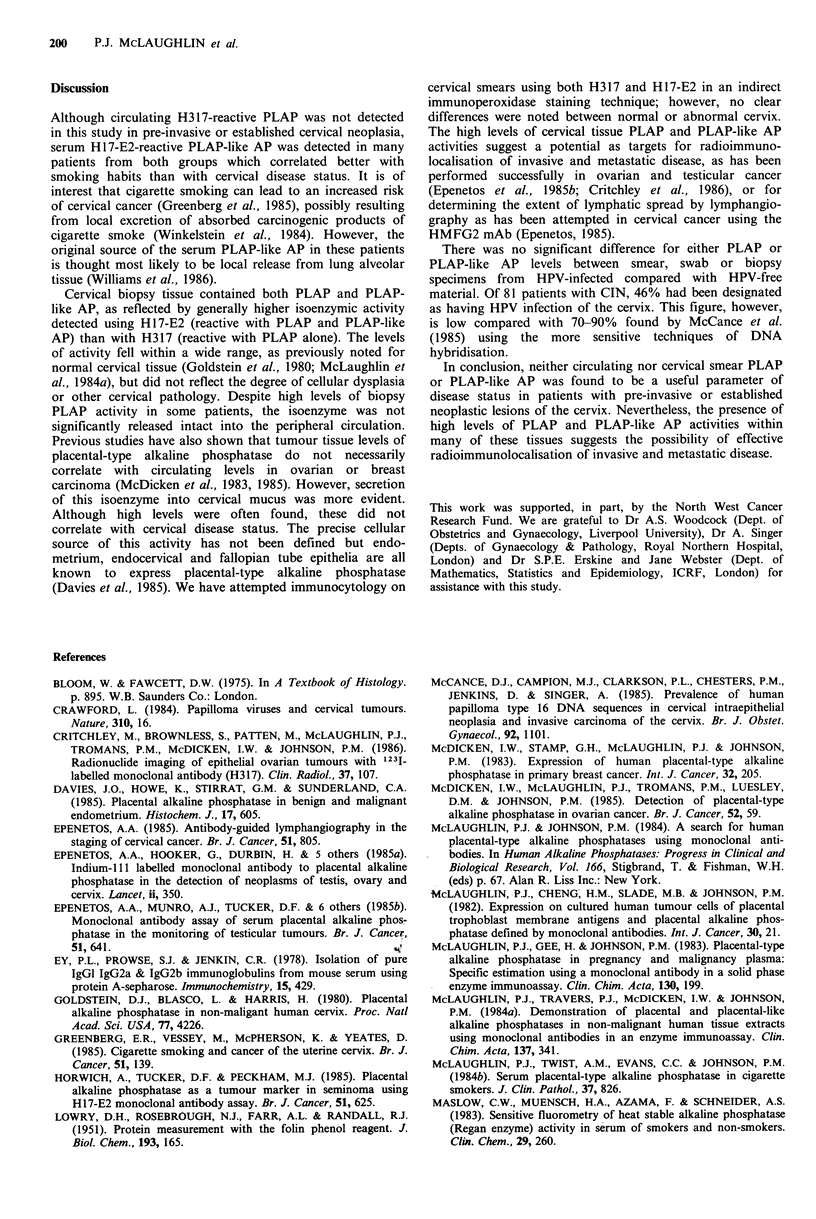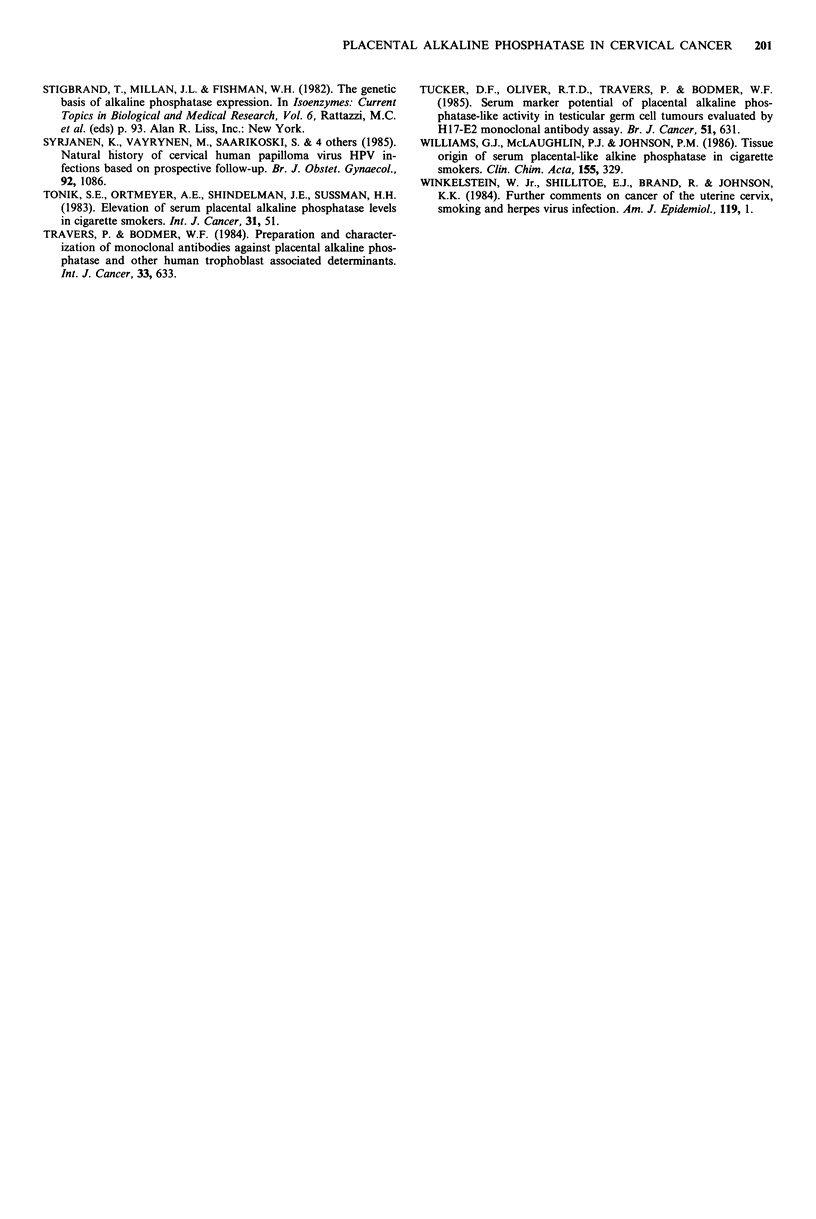# Placental-type alkaline phosphatase in cervical neoplasia.

**DOI:** 10.1038/bjc.1987.37

**Published:** 1987-02

**Authors:** P. J. McLaughlin, P. H. Warne, G. E. Hutchinson, P. M. Johnson, D. F. Tucker

## Abstract

Monoclonal antibodies reactive with placental-type alkaline phosphatase have formed the basis of methods for detection of this oncodevelopmental antigen in patients with pre-invasive and invasive cervical neoplasia, with or without evidence of papilloma virus infection. Disease-related elevations of placental-type alkaline phosphatase were not observed in patients' sera. Solubilised cervical smears or biopsy material, and cervical mucus swabs, often contained substantial amounts of this isoenzyme; however, there was no significant difference between any of the patient and control groups. Thus, serological and smear test assays for placental-type alkaline phosphatase were not useful in differential diagnosis of cervical lesions. However, its presence in most biopsy specimens, often at high levels, indicated possible application for in vivo radioimmunoimaging studies of invasive or metastatic cervical cancer.


Br. J. Cancer (1987), 55, 197–201 © The Macmillan Press Ltd., 1987

### Placental-type alkaline phosphatase in cervical neoplasia

P.J. McLaughlin[1], P.H. Warne[2], G.E. Hutchinson[1], P.M. Johnson[1] & D.F. Tucker[2]

[1]*Department of Immunology, University of Liverpool, P.O. Box 147, Liverpool L69 3BX and* [2]*Imperial Cancer Research Fund, P.O. Box 123, Lincoln's Inn Fields, London WC2A 3PX, UK.*

**Summary** Monoclonal antibodies reactive with placental-type alkaline phosphatase have formed the basis of methods for detection of this oncodevelopmental antigen in patients with pre-invasive and invasive cervical neoplasia, with or without evidence of papilloma virus infection. Disease-related elevations of placental-type alkaline phosphatase were not observed in patients' sera. Solubilised cervical smears or biopsy material, and cervical mucus swabs, often contained substantial amounts of this isoenzyme; however, there was no significant difference between any of the patient and control groups. Thus, serological and smear test assays for placental-type alkaline phosphatase were not useful in differential diagnosis of cervical lesions. However, its presence in most biopsy specimens, often at high levels, indicated possible application for *in vivo* radioimmunoimaging studies of invasive or metastatic cervical cancer.

The incidence of cervical cancer has increased markedly in younger women over the last two decades, in part due to venereal transmission of papilloma virus (HPV) infection (Crawford, 1984). It is not clear whether HPV infection can be a forerunner of cervical intraepithelial neoplasia (CIN), but progression of CIN into overt cancer may be related to concomitant infection by HPV (Syrjanen *et al.,* 1985). Screening for pre-invasive cervical carcinoma involves exhaustive examination of smears, and therefore simple rapid techniques for identifying pre-invasive cervical neoplasia are attractive to seek.

Potential markers for neoplastic cellular transformation are the oncodevelopmental antigens, such as placental-type alkaline phosphatase. This isoenzyme is a major glycoprotein of term placental trophoblast membranes, and is also expressed on some tumour cell lines including cervical carcinoma cells (McLaughlin *et al.,* 1982). Two major forms of placental-type alkaline phosphatase have been described, the 'placental' and 'placental-like' alkaline phosphatases (PLAP and PLAP-like AP, respectively). These can be distinguished by relative sensitivity to inhibition by L-leucine (Stigbrand *et al.,* 1982) and differential reactivity with certain monoclonal antibodies (mAbs) (McLaughlin & Johnson, 1984).

The development of sensitive and specific solid-phase enzyme capture immunoassays (EIA) utilising either the mAb H317 (reactive solely with PLAP) or the mAb H17-E2 (reactive with both PLAP and PLAP-like AP) has broadened investigations of this isoenzyme group in cancer (McLaughlin *et al.,* 1983; 1984*a*, *b*; Epenetos *et al.,* 1985*a*; Horwich *et al.,* 1985; Tucker *et al.,* 1985). For example, previous serological assays have been limited by detection of elevated isoenzyme levels in healthy individuals, particularly cigarette smokers (Maslow *et al.,* 1983; Tonik *et al.,* 1983). It is now clear that this smoking-associated enzyme is PLAP-like AP, rather than PLAP, and thus is unreactive in the H317-based assay (McLaughlin *et al.,* 1984*b*). Both H317 and H17-E2 have now been used to evaluate PLAP and PLAP-like AP in pre-invasive and established cervical neoplasia. Four approaches were developed: EIA assay of PLAP and PLAP-like AP in serum, solubilised cervical biopsy tissue material, cervical smears and cervical mucus swabs.

### Patients and methods

### *Patients*

The study groups for CIN were taken from a total of 48 women attending a colposcopy clinic at the Royal Liverpool Hospital, and 68 outpatients seen at the Royal Northern Hospital, London, because of abnormal cytology. Cervical punch biopsies were classified as normal, CIN I, CIN II or CIN III. These were further subdivided into those having evidence of HPV infection of the cervix (46%) and those appearing virus-free (54%), as assessed by macroscopic appearance of the cervix and light microscopic examination of smear and biopsy samples. Features suggestive of HPV infection, based on histological appearance, were the presence of keratinisation, papillomatosis, perinuclear vacuolation and observation of eosinophilic bodies within cells. Twelve patients from the Oxford region with established cancer of the cervix were also studied. These had squamous carcinoma ($n=9$), adenocarcinoma ($n=2$) or mixed squamous and adenocarcinoma ($n=1$); three of the carcinomas were Stage IA, five were Stage IB, three were IIA, and one was IIB.

### *Monoclonal antibodies (mAbs)*

The H317 and H17-E2 hybridomas had been produced following immunisation of mice with isolated human term placental syncytiotrophoblast microvilli (McLaughlin *et al.,* 1982, Travers & Bodmer, 1984). The secreted mAbs are both IgG1 and were purified from ascitic fluid by affinity chromatography with Sepharose-protein-A (Pharmacia) (Ey *et al.,* 1978). The H17-E2 mAb reacts with both PLAP and PLAP-like AP, whereas the H317 mAb reacts only with PLAP; neither mAb reacts with non-placental-type alkaline phosphatase isoenzymes (McLaughlin *et al.,* 1982, 1983; Travers & Bodmer, 1984).

### *Enzyme immunoassays (EIA)*

A solid-phase capture assay for PLAP in body fluids, based on the H317 mAb, has been described in detail (McLaughlin *et al.,* 1983; 1984*b*). The lower limit of detection is less than $0.1\,U\,l^{-1}$, and none of 120 healthy individuals had serum PLAP levels of $>0.1\,U\,l^{-1}$. The H17-E2 mAb has formed the basis of a similar EIA which measures both PLAP and PLAP-like AP (Tucker *et al.,* 1985). The lower limit of detection is $0.04\,U\,l^{-1}$, but occasional healthy individuals and most cigarette smokers have serum levels substantially greater than this (Tucker *et al.,* 1985) and which appears to be PLAP-like AP rather than PLAP (McLaughlin *et al.,* 1984*b*).

### *Solubilised tissue extracts*

Cervical biopsy samples (2–15 mg wet weight tissue) were suspended in 50 vol of PBS with 0.05% Tween 20, pH 7.4, and sonicated for a total of 2 min at 4°C. Residual fine particles were allowed to settle for 30 min and the super-

Correspondence: P.J. McLaughlin.
Received 25 July 1986; and in revised form, 3 October 1986.

natants assayed for placental-type alkaline phosphatases by EIA with a lower limit of detection of $5\,U\,kg^{-1}$ tissue.

*Cervical smear assay*

Spatula smear material was solubilised with 2 ml 0.2% sodium deoxycholate in 0.1 M phosphate buffer, pH 8.5. The suspension was centrifuged at 900*g* for 15 min and the supernatant assayed for both protein (Lowry *et al.,* 1951) and placental-type alkaline phosphatases by EIA with a lower limit of detection of $0.5\,U\,g^{-1}$ protein.

*Cervical swab assay*

A swab of endocervical mucus was placed in 3 ml PBS, pH 7.4, and agitated to disperse the mucus. This material was then assayed for placental-type alkaline phosphatases by EIA. Results were expressed as $U\,l^{-1}$.

Cervical mucus samples from normal subjects were collected at a family planning clinic in Sheffield by Dr Nigel Saunders, Department of Obstetrics & Gynaecology, Northern General Hospital, Sheffield.

### Results

*Serological assay*

Circulating H317-reactive PLAP ($>0.1\,U\,l^{-1}$) was not detected in any of 22 CIN patients or 12 patients with established cervical cancer, with or without evidence of HPV infection. However, 12 of these sera did contain H17-E2-reactive PLAP-like AP; this correlated with the expected pattern for smoking-associated circulating PLAP-like AP, since 11 of these 12 patients were cigarette smokers.

*Cervical tissue biopsies*

Of biopsies taken, 31/35 (89%) had detectable placental-type alkaline phosphatase activity in solubilised tissue extracts which was reactive with both H317 and H17-E2 (Figure 1). The H17-E2 reactivity was greater than H317 reactivity in most cases, reflecting co-expression of both PLAP and PLAP-like AP. There was no correlation between placental-type alkaline phosphatase levels and degree of dysplasia or presence of HPV infection of the cervix.

*Cervical smears*

Solubilised smears contained placental-type alkaline phosphatase activity detectable using both H317 and H17-E2 in 14/29 (48%) cases studied (Figure 2). There was reactivity with H17-E2 but not H317 in a further 7 cases (24%), and smears from 8 cases (28%) contained no detectable placental-type alkaline phosphatase reactivity by either assay. There was no difference in distribution of isoenzyme activities between any of the patient groups.

Both biopsy material and smears were available from 16 patients. Patients with high tissue biopsy placental-type alkaline phosphatase activities often also had high isoenzymic activity in solubilised smears, although this was not statistically significant by product–moment correlation coefficient analysis.

*Cervical swabs*

There was a wide range for placental-type alkaline phosphatase activity in swab material (Figure 3) similar to that for the smear assay. Over half the samples (51/93, 55%) were reactive in both the H317-based and H17-E2-based assays, and only 29/93 (31%) were negative in both assays. Six of 12 (50%) patients with established cervical cancer had placental-type alkaline phosphatase in cervical mucus detectable with both H317 and H17-E2. This was a similar proportion to that found in CIN patients or normal (no CIN) controls (Figure 3).

The nature of the mucus and its rate of production change around the time of ovulation (Bloom & Fawcett, 1975), although cervical swab placental-type alkaline phosphatase levels compared at different stages of the menstrual cycle, showed no significant differences.

**Figure 1** Placental-type alkaline phosphatase activity in cervical biopsy material. ○: H17-E2-reactive activity; ●: H317-reactive activity; HPV: evidence of HPV infection.

**Figure 2** Placental-type alkaline phosphatase activity in cervical smear material. ○: H17-E2-reactive activity; ●: H317-reactive activity; HPV: evidence of HPV infection.

**Figure 3** Placental-type alkaline phosphatase activity in cervical mucus. ○: H17-E2-reactive activity; ●: H317-reactive activity; HPV: evidence of HPV infection.

### Discussion

Although circulating H317-reactive PLAP was not detected in this study in pre-invasive or established cervical neoplasia, serum H17-E2-reactive PLAP-like AP was detected in many patients from both groups which correlated better with smoking habits than with cervical disease status. It is of interest that cigarette smoking can lead to an increased risk of cervical cancer (Greenberg *et al.*, 1985), possibly resulting from local excretion of absorbed carcinogenic products of cigarette smoke (Winkelstein *et al.*, 1984). However, the original source of the serum PLAP-like AP in these patients is thought most likely to be local release from lung alveolar tissue (Williams *et al.*, 1986).

Cervical biopsy tissue contained both PLAP and PLAP-like AP, as reflected by generally higher isoenzymic activity detected using H17-E2 (reactive with PLAP and PLAP-like AP) than with H317 (reactive with PLAP alone). The levels of activity fell within a wide range, as previously noted for normal cervical tissue (Goldstein *et al.*, 1980; McLaughlin *et al.*, 1984*a*), but did not reflect the degree of cellular dysplasia or other cervical pathology. Despite high levels of biopsy PLAP activity in some patients, the isoenzyme was not significantly released intact into the peripheral circulation. Previous studies have also shown that tumour tissue levels of placental-type alkaline phosphatase do not necessarily correlate with circulating levels in ovarian or breast carcinoma (McDicken *et al.*, 1983, 1985). However, secretion of this isoenzyme into cervical mucus was more evident. Although high levels were often found, these did not correlate with cervical disease status. The precise cellular source of this activity has not been defined but endometrium, endocervical and fallopian tube epithelia are all known to express placental-type alkaline phosphatase (Davies *et al.*, 1985). We have attempted immunocytology on cervical smears using both H317 and H17-E2 in an indirect immunoperoxidase staining technique; however, no clear differences were noted between normal or abnormal cervix. The high levels of cervical tissue PLAP and PLAP-like AP activities suggest a potential as targets for radioimmunolocalisation of invasive and metastatic disease, as has been performed successfully in ovarian and testicular cancer (Epenetos *et al.*, 1985*b*; Critchley *et al.*, 1986), or for determining the extent of lymphatic spread by lymphangiography as has been attempted in cervical cancer using the HMFG2 mAb (Epenetos, 1985).

There was no significant difference for either PLAP or PLAP-like AP levels between smear, swab or biopsy specimens from HPV-infected compared with HPV-free material. Of 81 patients with CIN, 46% had been designated as having HPV infection of the cervix. This figure, however, is low compared with 70–90% found by McCance *et al.* (1985) using the more sensitive techniques of DNA hybridisation.

In conclusion, neither circulating nor cervical smear PLAP or PLAP-like AP was found to be a useful parameter of disease status in patients with pre-invasive or established neoplastic lesions of the cervix. Nevertheless, the presence of high levels of PLAP and PLAP-like AP activities within many of these tissues suggests the possibility of effective radioimmunolocalisation of invasive and metastatic disease.

This work was supported, in part, by the North West Cancer Research Fund. We are grateful to Dr A.S. Woodcock (Dept. of Obstetrics and Gynaecology, Liverpool University), Dr A. Singer (Depts. of Gynaecology & Pathology, Royal Northern Hospital, London) and Dr S.P.E. Erskine and Jane Webster (Dept. of Mathematics, Statistics and Epidemiology, ICRF, London) for assistance with this study.

### References

BLOOM, W. & FAWCETT, D.W. (1975). In *A Textbook of Histology*. p. 895. W.B. Saunders Co.: London.

CRAWFORD, L. (1984). Papilloma viruses and cervical tumours. *Nature*, **310**, 16.

CRITCHLEY, M., BROWNLESS, S., PATTEN, M., McLAUGHLIN, P.J., TROMANS, P.M., McDICKEN, I.W. & JOHNSON, P.M. (1986). Radionuclide imaging of epithelial ovarian tumours with $^{123}$I-labelled monoclonal antibody (H317). *Clin. Radiol.*, **37**, 107.

DAVIES, J.O., HOWE, K., STIRRAT, G.M. & SUNDERLAND, C.A. (1985). Placental alkaline phosphatase in benign and malignant endometrium. *Histochem. J.*, **17**, 605.

EPENETOS, A.A. (1985). Antibody-guided lymphangiography in the staging of cervical cancer. *Br. J. Cancer*, **51**, 805.

EPENETOS, A.A., HOOKER, G., DURBIN, H. & 5 others (1985*a*). Indium-111 labelled monoclonal antibody to placental alkaline phosphatase in the detection of neoplasms of testis, ovary and cervix. *Lancet*, **ii**, 350.

EPENETOS, A.A., MUNRO, A.J., TUCKER, D.F. & 6 others (1985*b*). Monoclonal antibody assay of serum placental alkaline phosphatase in the monitoring of testicular tumours. *Br. J. Cancer*, **51**, 641.

EY, P.L., PROWSE, S.J. & JENKIN, C.R. (1978). Isolation of pure IgGl IgG2a & IgG2b immunoglobulins from mouse serum using protein A-sepharose. *Immunochemistry*, **15**, 429.

GOLDSTEIN, D.J., BLASCO, L. & HARRIS, H. (1980). Placental alkaline phosphatase in non-maligant human cervix. *Proc. Natl Acad. Sci. USA*, **77**, 4226.

GREENBERG, E.R., VESSEY, M., McPHERSON, K. & YEATES, D. (1985). Cigarette smoking and cancer of the uterine cervix. *Br. J. Cancer*, **51**, 139.

HORWICH, A., TUCKER, D.F. & PECKHAM, M.J. (1985). Placental alkaline phosphatase as a tumour marker in seminoma using H17-E2 monoclonal antibody assay. *Br. J. Cancer*, **51**, 625.

LOWRY, D.H., ROSEBROUGH, N.J., FARR, A.L. & RANDALL, R.J. (1951). Protein measurement with the folin phenol reagent. *J. Biol. Chem.*, **193**, 165.

McCANCE, D.J., CAMPION, M.J., CLARKSON, P.L., CHESTERS, P.M., JENKINS, D. & SINGER, A. (1985). Prevalence of human papilloma type 16 DNA sequences in cervical intraepithelial neoplasia and invasive carcinoma of the cervix. *Br. J. Obstet. Gynaecol.*, **92**, 1101.

McDICKEN, I.W., STAMP, G.H., McLAUGHLIN, P.J. & JOHNSON, P.M. (1983). Expression of human placental-type alkaline phosphatase in primary breast cancer. *Int. J. Cancer*, **32**, 205.

McDICKEN, I.W., McLAUGHLIN, P.J., TROMANS, P.M., LUESLEY, D.M. & JOHNSON, P.M. (1985). Detection of placental-type alkaline phosphatase in ovarian cancer. *Br. J. Cancer*, **52**, 59.

McLAUGHLIN, P.J. & JOHNSON, P.M. (1984). A search for human placental-type alkaline phosphatases using monoclonal antibodies. In *Human Alkaline Phosphatases: Progress in Clinical and Biological Research, Vol. 166*, Stigbrand, T. & Fishman, W.H. (eds) p. 67. Alan R. Liss Inc.: New York.

McLAUGHLIN, P.J., CHENG, H.M., SLADE, M.B. & JOHNSON, P.M. (1982). Expression on cultured human tumour cells of placental trophoblast membrane antigens and placental alkaline phosphatase defined by monoclonal antibodies. *Int. J. Cancer*, **30**, 21.

McLAUGHLIN, P.J., GEE, H. & JOHNSON, P.M. (1983). Placental-type alkaline phosphatase in pregnancy and malignancy plasma: Specific estimation using a monoclonal antibody in a solid phase enzyme immunoassay. *Clin. Chim. Acta*, **130**, 199.

McLAUGHLIN, P.J., TRAVERS, P.J., McDICKEN, I.W. & JOHNSON, P.M. (1984*a*). Demonstration of placental and placental-like alkaline phosphatases in non-malignant human tissue extracts using monoclonal antibodies in an enzyme immunoassay. *Clin. Chim. Acta*, **137**, 341.

McLAUGHLIN, P.J., TWIST, A.M., EVANS, C.C. & JOHNSON, P.M. (1984*b*). Serum placental-type alkaline phosphatase in cigarette smokers. *J. Clin. Pathol.*, **37**, 826.

MASLOW, C.W., MUENSCH, H.A., AZAMA, F. & SCHNEIDER, A.S. (1983). Sensitive fluorometry of heat stable alkaline phosphatase (Regan enzyme) activity in serum of smokers and non-smokers. *Clin. Chem.*, **29**, 260.

STIGBRAND, T., MILLAN, J.L. & FISHMAN, W.H. (1982). The genetic basis of alkaline phosphatase expression. In *Isoenzymes: Current Topics in Biological and Medical Research, Vol. 6,* Rattazzi, M.C. *et al.* (eds) p. 93. Alan R. Liss, Inc.: New York.

SYRJANEN, K., VAYRYNEN, M., SAARIKOSKI, S. & 4 others (1985). Natural history of cervical human papilloma virus HPV infections based on prospective follow-up. *Br. J. Obstet. Gynaecol.*, **92,** 1086.

TONIK, S.E., ORTMEYER, A.E., SHINDELMAN, J.E., SUSSMAN, H.H. (1983). Elevation of serum placental alkaline phosphatase levels in cigarette smokers. *Int. J. Cancer,* **31,** 51.

TRAVERS, P. & BODMER, W.F. (1984). Preparation and characterization of monoclonal antibodies against placental alkaline phosphatase and other human trophoblast associated determinants. *Int. J. Cancer,* **33,** 633.

TUCKER, D.F., OLIVER, R.T.D., TRAVERS, P. & BODMER, W.F. (1985). Serum marker potential of placental alkaline phosphatase-like activity in testicular germ cell tumours evaluated by H17-E2 monoclonal antibody assay. *Br. J. Cancer,* **51,** 631.

WILLIAMS, G.J., McLAUGHLIN, P.J. & JOHNSON, P.M. (1986). Tissue origin of serum placental-like alkine phosphatase in cigarette smokers. *Clin. Chim. Acta,* **155,** 329.

WINKELSTEIN, W. Jr., SHILLITOE, E.J., BRAND, R. & JOHNSON, K.K. (1984). Further comments on cancer of the uterine cervix, smoking and herpes virus infection. *Am. J. Epidemiol.*, **119,** 1.